# Pravastatin Promotes Endothelial Colony-Forming Cell Function, Angiogenic Signaling and Protein Expression In Vitro

**DOI:** 10.3390/jcm10020183

**Published:** 2021-01-06

**Authors:** Nadia Meyer, Lars Brodowski, Katja Richter, Constantin S. von Kaisenberg, Bianca Schröder-Heurich, Frauke von Versen-Höynck

**Affiliations:** 1 Gynecology Research Unit, Hannover Medical School, Carl-Neuberg-Strasse 1, D-30625 Hannover, Germany; Meyer.Nadia@mh-hannover.de (N.M.); Brodowski.Lars@mh-hannover.de (L.B.); Richter.Katja@mh-hannover.de (K.R.); Schroeder-Heurich.Bianca@mh-hannover.de (B.S.-H.); 2 Department of Obstetrics and Gynecology, Hannover Medical School, Carl-Neuberg-Strasse 1, D-30625 Hannover, Germany; vonKaisenberg.Constantin@mh-hannover.de

**Keywords:** endothelial colony-forming cells, endothelial progenitor cells, endothelial dysfunction, pravastatin, statins, cell therapy, cardiovascular disease, preeclampsia, angiogenesis, vascular repair

## Abstract

Endothelial dysfunction is a primary feature of several cardiovascular diseases. Endothelial colony-forming cells (ECFCs) represent a highly proliferative subtype of endothelial progenitor cells (EPCs), which are involved in neovascularization and vascular repair. Statins are known to improve the outcome of cardiovascular diseases via pleiotropic effects. We hypothesized that treatment with the 3-hydroxy-3-methyl-glutaryl–coenzyme A (HMG-CoA) reductase inhibitor pravastatin increases ECFCs’ functional capacities and regulates the expression of proteins which modulate endothelial health in a favourable manner. Umbilical cord blood derived ECFCs were incubated with different concentrations of pravastatin with or without mevalonate, a key intermediate in cholesterol synthesis. Functional capacities such as migration, proliferation and tube formation were addressed in corresponding in vitro assays. mRNA and protein levels or phosphorylation of protein kinase B (AKT), endothelial nitric oxide synthase (eNOS), heme oxygenase-1 (HO-1), vascular endothelial growth factor A (VEGF-A), placental growth factor (PlGF), soluble fms-like tyrosine kinase-1 (sFlt-1) and endoglin (Eng) were analyzed by real time PCR or immunoblot, respectively. Proliferation, migration and tube formation of ECFCs were enhanced after pravastatin treatment, and AKT- and eNOS-phosphorylation were augmented. Further, expression levels of HO-1, VEGF-A and PlGF were increased, whereas expression levels of sFlt-1 and Eng were decreased. Pravastatin induced effects were reversible by the addition of mevalonate. Pravastatin induces beneficial effects on ECFC function, angiogenic signaling and protein expression. These effects may contribute to understand the pleiotropic function of statins as well as to provide a promising option to improve ECFCs’ condition in cell therapy in order to ameliorate endothelial dysfunction.

## 1. Introduction

Cardiovascular diseases (CVD) are considered a major healthcare problem and represent the leading cause of mortality and morbidity worldwide [1,2]. They comprise a heterogeneous group of disorders that affect the heart and blood vessels and are often based on endothelial dysfunction [3]. Coronary artery disease as one of the most frequent manifestations is associated with the stenosis or occlusion of vessels [4]. The therapy mainly consists of vascular regenerative surgery and pharmacological therapy for alleviation, but not all patients benefit from current treatment options. Novel therapeutic approaches not only addressing the symptoms, but also the underlying pathological processes promoting neovascularization and vascular repair need to be developed [5,6].

Endothelial progenitor cells (EPCs) have been studied extensively since they were first described and isolated by Asahara et al. in 1997 [7]. These bone-marrow derived cells circulate in peripheral blood and are highly enriched in umbilical cord blood [8,9]. EPCs are impaired in number and functional capacity in heart failure [10,11,12], hypertension [13], diabetes mellitus [14,15,16] and gestational diseases, e.g., preeclampsia [17,18], and inversely correlate with risk factors for coronary artery disease [19]. Therefore, they are now considered to be one of the strongest biomarkers to evaluate endothelial dysfunction [20,21]. 

Endothelial colony-forming cells (ECFCs), a subgroup of EPCs, are known for their high proliferative and clonal capacity and play an important role in angiogenesis and vascular repair [22,23]. They contribute to endothelial integrity not only by incorporation into damaged vessel walls and thereby stabilizing them, but also through paracrine effects via cytokine release [24,25]. Recently, ECFCs have been identified as a promising model for cell-based therapy and have been addressed as a therapeutic target in several studies [21,26,27,28,29]. However, the results are still not satisfying and further approaches must be explored to improve ECFCs’ survival and functional abilities. 

Statins are a class of drugs that reduce morbidity and mortality in patients at high cardiovascular risk [30,31]. They inhibit the 3-hydroxy-3-methyl-glutaryl–coenzyme A (HMG-CoA) reductase that catalyzes the mevalonate synthesis, the limiting step in cholesterol biosynthesis, and are widely used as a lipid-regulating drug [32,33]. Statins exhibit several other beneficial pleiotropic effects, e.g., the regulation of endothelial function by modulating the immune system, reducing vascular inflammation, decreasing platelet aggregation, providing anti-atherothrombotic properties and regulating the expression of molecules involved in endothelial cell function [32,34,35,36,37,38,39,40,41].

Protein kinase B (AKT) is a serine/threonine protein kinase and acts as multifunctional intracellular regulator of cell growth, metabolism and survival, whereas endothelial nitric oxide synthase (eNOS) is known to be a downstream target in the AKT pathway and is mainly responsible for NO synthesis and, therefore, for vascular relaxation [42,43]. There has been former evidence that statins may also increase the release of pro-angiogenic molecules, e.g., vascular endothelial growth factor A (VEGF-A) or placental growth factor (PlGF), and decrease anti-angiogenic factors, e.g., soluble fms-like tyrosine kinase-1 (sFlt-1) or soluble endoglin (sEng), from different cells [44,45,46,47]. A disbalance of these molecules towards increased levels of anti-angiogenic factors is known in preeclampsia, which is characterized by generalized endothelial dysfunction and hypertension in pregnancy [48,49,50].

In light of the potential of ECFCs in cell-based therapies, the present study systematically explores on functional and mechanistic levels whether pravastatin, a hydrophilic statin variant, modulates ECFCs’ functional capacity and activates vasoprotective mechanisms of ECFCs.

## 2. Material and Methods

### 2.1. ECFC Isolation, Culture and Characterization 

Umbilical cord blood from six healthy, uncomplicated singleton pregnancies was collected immediately after delivery. All women included were normotensive, without proteinuria throughout gestation, and had healthy babies at term. This research was approved by the local Ethic Committee of Hannover Medical School (approval no. 1443-2012) and written informed consent was provided by each participant.

ECFCs were isolated as previously described by Grundmann et al. [51]. Umbilical cord blood was centrifuged and plasma was removed for storage. After density gradient centrifugation, the cord blood mononuclear cell (CBMC) fraction was plated in endothelial growth medium 2 (EGM-2) consisting of endothelial basal medium (EBM-2, Lonza, Basel, Switzerland, for isolation and PromoCell, Heidelberg, Germany, for experiments), supplemented with supplier provided supplements and additional 10% fetal bovine serum (FBS, Harvard Bioscience, Holliston, MA, USA) and 1% penicillin/streptomycin (P/S, Bio&Sell, Feucht, Nürnberg, Germany) at a density of up to 1 × 10^7^ cells/well on collagen coated 6-well plates (BioCoat; Corning, Corning, NY, USA) and incubated at 37 °C, 5% CO_2_ supply. After 10 to 20 days, first appearance of ECFC colonies was noted as well circumscribed monolayers of rapidly proliferating, cobblestone-appearing cells and characterized by flow cytometry with appropriate antibodies (CD31 (130-117-390; BD Biosciences, San Jose, CA, USA), CD45 (555483; BD Biosciences), and CD133 (130-090-826; Miltenyi Biotec, Bergisch Gladbach, Germany)) and the corresponding isotype controls (BD Biosciences, Miltenyi Biotec) [51,52]. ECFCs from biologically different individuals were kept separately in culture and for all experiments which were run in cell culture passages 4–6.

### 2.2. Chemotaxis Assay

To analyze ECFCs’ ability of directional cell migration in the presence or absence of pravastatin (Merck, Darmstadt, Germany), we performed a modified Boyden chamber assay. Transwell inserts with an 8 µm microporous membrane (ThinCerts; Greiner, Kremsmünster, Austria) were placed in a 12-well-plate. Then, 5 × 10^4^ ECFCs from 3–4 biologically distinct lines were seeded into the upper side of the chamber in serum-free EBM-2 medium with 1% P/S but without other supplements, and 10% FBS was used as chemoattractant in the lower side of the chamber. Different concentrations (2–2000 µM) of pravastatin were added to both sides of the chamber in the presence or absence of 200 µM mevalonate (Sigma Aldrich, Steinheim, Germany). The cells were allowed to migrate for 4 h until the inserts were removed and the non-migrated cells were detached from the upper surface of the membrane with a cotton bud. The inserts were placed in fixing solution as described above, followed by two washing steps with PBS. Afterwards, the migrated cells on the lower side of the membrane were counterstained with 4′,6-diamidino-2-phenylindole (DAPI, Thermo Fisher Scientific, Waltham, MA, USA), followed by mounting in antifade fluorescence mounting medium (ProLongGold; Thermo Fisher Scientific). Image acquisition was realized with a Leica DMI 6000 B microscope (Leica, Wetzlar, Germany). Five pictures per membrane were randomly taken and DAPI-stained cells were counted with ImageJ 1.50b (National Institutes of Health).

### 2.3. Scratch Wound Healing Assay

A scratch wound healing assay was used to assess ECFC’s ability to migrate in order to close a wound. ECFCs (5 × 10^4^ cells) from 4 biologically distinct lines were seeded on gelatine (Sigma-Aldrich) coated wells of 6-well culture plates with EGM-2 containing 10% FBS and 1% P/S. After cells reached optical confluence, they were kept in culture for an additional 24 h. The cell monolayers were scratched with a sterile P1000 pipette tip to create a wound, media was aspirated and, after washing the cells with PBS, replaced with treatment media (EBM-2 without supplements, 2.5% FBS and 1% P/S) containing different doses (2–2000 µM) of pravastatin with or without an additional 200 µM mevalonate. Each treatment was performed in duplicate wells. Phase contrast microscopic images were immediately obtained after scratching and then again after 18 h. Non-populated scratch areas were quantified by ImageJ 1.50b and subtracted to obtain the remigrated area.

### 2.4. In Vitro Angiogenesis Assay

The capacity of ECFCs to form capillary tubule-like networks was tested by seeding 14,000 ECFCs/well from 4 biologically distinct lines in triplicates in 96-well plates pre-coated with 30 µL growth factor reduced Matrigel (BD Biosciences). The cells were incubated for 6 h with different concentrations (2–2000 µM) of pravastatin with or without 200 µM mevalonate in EBM-2 with 1% P/S without growth supplements. Phase contrast microscopic images were obtained at 20x magnification with a Leica DMI 6000 B microscope. The total tubule length and number of branch points in each visual field were measured using ImageJ 1.50b. Branch points were defined as nodes with connections to at least 3 tubes.

### 2.5. Proliferation Assay

Cell proliferation was measured with the xCelligence Real-Time Cell Analyzer (Roche, Basel, Switzerland). The xCelligence system allows us to monitor cell proliferation continuously in real time. The electrical impedance caused by the adherent cells is converted into cell indices by the xCelligence software (v.1.2.1, Roche, Basel, Switzerland). A total of 2000 to 3000 cells from 4–5 biologically distinct ECFC lines were seeded in quadruplicates in EGM-2 with 8% FBS and 1% P/S onto a gold-coated E-Plate View 96-well plate (Roche) and then placed into the Real-Time Cell Analyzer SP station, positioned in a 37 °C incubator with 5% CO_2_ supply. After reaching a stable cell index, different concentrations (2–2000 µM) of pravastatin with or without 200 µM mevalonate were added. Cell growth was continuously monitored for 72 h after pravastatin addition.

### 2.6. Apoptosis Assay 

Flow cytometry analysis was performed to measure apoptosis. After 48 h of treatment with different concentrations of pravastatin (2–2000 µM) or mevalonate (200 µM) in EGM-2 with 8% FBS and 1% P/S, ECFCs from 4–5 biologically distinct lines were harvested with 0.05% trypsin/EDTA and washed with flow cytometry buffer (PBS, 2% FBS). Then, 1 × 10^5^ cells were blocked with Intraglobin (5 mg/mL; Gamunex 10%, Grifols, Frankfurt am Main, Germany) for 1 min, followed by incubation with an Annexin V antibody (#640906, Biolegend, San Diego, California, USA) or isotype control at 4 °C for 30 min. UV radiation for 30 min was used as positive control for apoptosis for gating strategy. Propidiumiodide (PI, Sigma Aldrich) was added one min prior to measurement on a BD FACS Calibur Flow Cytometer (BD Biosciences), and results were analyzed using FlowJo X Software v.10 (Tree Star, FlowJo ILC; Ashland, OR, USA).

### 2.7. Isolation of Proteins and Immunoblotting:

For analyses of proteins, ECFCs from 2–5 biologically distinct cell lines were grown to 80–90% confluence in 10 cm dishes (Sarstedt, Nümbrecht, Germany) and treated with 200 µM pravastatin for 2 h (pAKT, peNOS analysis) or 24 h (HO-1, VEGF-A analysis). Mevalonate (200 µM) was applied simultaneously to block potential modifications. ECFCs were detached with trypsin-EDTA, washed with PBS and lysed with lysis buffer as previously described. [51]. Protein concentration was determined by Bradford protein assay [53]. Proteins were separated by electrophoresis on SDS-polyacrylamide gels and transferred to nitrocellulose membranes (GE Healthcare, Waukesha, WI, USA). After blocking for 1 h with 1% Roti-Block (Carl Roth, Karlsruhe, Germany) in H_2_O, the membrane was incubated overnight at 4 °C with appropriate antibodies (1:1000 pAKT S473 and 1:1000 AKT (9271S and 9272S, Cell Signaling, Danvers, MA, USA), 1:3000 anti–beta-actin (A54410, Sigma-Aldrich, St. Louis, MO, USA), 1:3000 anti-alpha-tubulin (sc32293; Santa Cruz Biotechnology, Dallas, TX, USA), 1:1000 HO-1 (ADI-SPA-895, Enzo Life Sciences, Farmingdale, NY, USA), 1:1000 peNOS S1177, 1:1000 eNOS (sc81510, Santa Cruz; ab5589, Abcam, Cambridge, GB), and 1:500 VEGF-A (ab157160, Abcam)). After washing three times with PBS-T (0.1% *v*/*v*), the secondary antibody was added (1:3000 (or 1:5000 for beta-actin and alpha-tubulin) goat anti-mouse or 1:3000 goat anti-rabbit horseradish peroxidase; GE Healthcare) in 1% Roti-Block for 2 h at room temperature. Visualization of immunoblot bands was performed by using ECL chemiluminescence (Thermo Fisher Scientific, Waltham, MA, USA) and analyzed with ImageJ 1.50b.

### 2.8. Quantitative Real-Time PCR (qRT-PCR)

ECFCs from 3–5 biologically distinct lines were treated with 20 or 200 µM pravastatin in the presence or absence of 200 µM mevalonate for 24 h. RNA was isolated by using the RNeasy Plus Mini Kit (Qiagen, Hilden, Germany). For the cDNA synthesis, RNA was diluted with diethylpyrocarbonate (DEPC)-treated water and denatured at 68 °C for 10 min in a thermocycler (PTC 200, Biozym Scientific GmbH, Hessisch Oldendorf, Germany). Then, High Capacity cDNA Reverse transcription (RT) master mix was added, containing RT buffer, RT random primer, deoxyribonucleoside triphosphate (dNTP) mix (100 mM), MultiScribeTMReverse transcriptase (Applied Biosystems, Waltham, MA, USA) and DEPC-treated water. In each case, 1.5 µL cDNA and 10.5 µL master mix (FastStart Universal SYBR Green, Roche, Basel, Switzerland) were pipetted into the appropriate strip tubes (0.1 mL). Real-time PCR was performed on a Rotor-Gene 6000 (Qiagen) for 40 cycles. For each treatment, runs were performed in triplicates. The primer sequences used to determine mRNA levels of VEGF-A, PlGF, sFlt-1, Eng and HO-1 are described in Table 1. Ct values were automatically generated, and relative quantification of gene expression was calculated by standard ΔCt method using the expression of 18S rRNA as reference.

### 2.9. Statistical Analysis

Statistical analysis was performed after testing for normality distribution by the Shapiro–Wilk or D’Agostino normality test. Student’s unpaired *t* test, Mann–Whitney test, Kruskal–Wallis test, one sample *t* test or Wilcoxon’s signed-rank test were used as appropriate. Experimental data are presented as mean and standard error. All replicates (*n*) from each experiment were analyzed with Prism 9 (GraphPad Software, La Jolla, CA, USA). A significant deviation is indicated by values of *p* < 0.05, *p* < 0.01, *p* < 0.001. 

## 3. Results

### 3.1. Pravastatin Increases ECFC Migration

**Figure 1:**
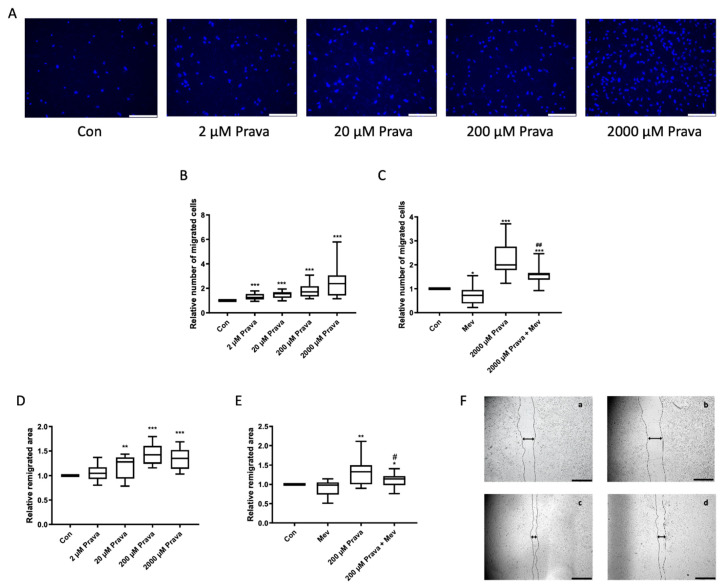
Pravastatin increased endothelial colony-forming cells’ (ECFCs’) migration. (**A**) Representative images of 4′,6-diamidino-2-phenylindole (DAPI)-stained migrated ECFCs treated with pravastatin (2, 20, 200 and 2000 µM) in a chemotaxis assay, scale bar 150 µm. (**B**) ECFCs showed a significantly higher directional migration in the presence of pravastatin after 4 h. (**C**) Mevalonate (200 µM) decreased directional migration and, in combination with pravastatin (2000 µM), significantly decreased the pravastatin induced increase in directional migration. Further representative images can be found in Supplemental Figure S1. Numbers of DAPI-stained migrated cells on the lower side of the membranes were counted in each picture, *n* = 15–20. (**D**) Pravastatin treatment (2, 20, 200 and 2000 µM) of ECFCs enhanced wound closure assessed as remigrated area after 18 h compared to control. (**E**) Mevalonate (200 µM) alone had no significant effect on ECFCs’ migration, but its addition to pravastatin (200 µM) reduced the pravastatin effect significantly. (**F**) Representative images of monolayers with scratch wounds at 18 h of incubation with culture medium only (a), 200 µM mevalonate (b), 200 µM pravastatin (c) and combination of 200 µM pravastatin and 200 µM mevalonate (d), scale bar 1000 µm. Cell-free area after 18 h was subtracted from cell-free area at start to calculate remigrated area. *n* = 14–16. Con, control; Prava, pravastatin; Mev, mevalonate. * *p* < 0.05, ** *p* < 0.01, *** *p* < 0.001 compared to control, # *p* < 0.05 compared to 200 µM pravastatin, ## p < 0.01 compared to 2000 µM pravastatin; (**B**–**E**) control group set as 1.

The directional migration of single cells is promoted by chemotaxis and was tested using a modified boyden chamber assay. Therefore, we explored how directional cell migration is impacted by different concentrations of pravastatin and if the effect can be revised by the addition of mevalonate, the product of HMG-CoA reductase which is inhibited by pravastatin. The chemotaxis ability of ECFCs treated with pravastatin was significantly higher compared with the control and increased with higher doses (relative number of migrated cells: 2 µM: 1.30 ± 0.05; 20 µM: 1.46 ± 0.07; 200 µM: 1.80 ± 0.11; 2000 µM: 2.64 ± 0.32; *n* = 20, *p* < 0.001), Figure 1A,B. The addition of mevalonate (200 µM) reduced the pravastatin effect significantly but not completely (relative number of migrated cells: 1.61 ± 0.10, *n* = 15, *p* < 0.001 compared to control and *p* = 0.003 compared to 2000 µM: 2.24 ± 0.17), whereas mevalonate alone caused a slight decrease in directional migration (0.73 ± 0.11, *n* = 15, *p* = 0.02), Figure 1C, Supplemental Figure S1. As pravastatin has been shown to increase the directional migration of ECFCs towards a chemoattractant, we further addressed if pravastatin also influences ECFC remigration of a scratch wound in a former monolayer. Wound closure was significantly higher in the presence of pravastatin (relative remigrated area: 2 µM: 1.05 ± 0.05, *p* = 0.30; 20 µM: 1.19 ± 0.06, *p* = 0.007; 200 µM: 1.43 ± 0.06, *p* < 0.001; 2000 µM: 1.35 ± 0.06, *p* < 0.001, *n* = 14), Figure 1D. Mevalonate alone had no significant effect on ECFC wound closure (0.90 ± 0.05, *n* = 16, *p* = 0.16) but lowered the beneficial effect of pravastatin (200 µM) significantly (1.11 ± 0.04, *p* = 0.02 compared to control and *p* = 0.03 compared to 200 µM pravastatin: 1.34 ± 0.09, *n* = 16), Figure 1E,F.

### 3.2. Pravastatin Enhances ECFC Angiogenic Capacity

**Figure 2:**
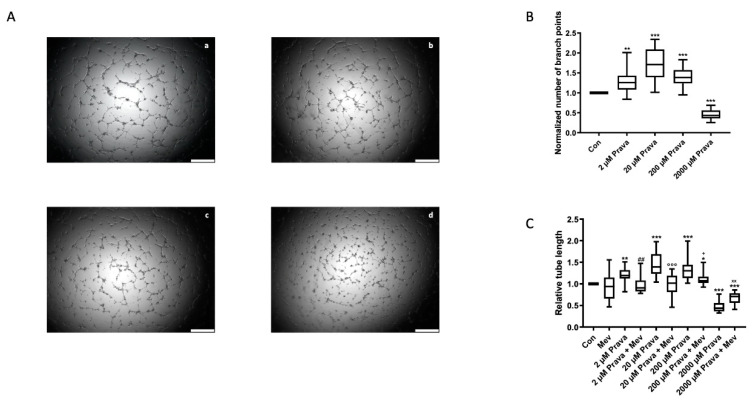
Pravastatin enhanced ECFCs’ angiogenesis. (**A**) Representative images from ECFCs treated with medium only (a), 20 µM (b), 200 µM (c) or 2000 µM (d) pravastatin for 6 h, scale bar 500 µm. (**B**) Pravastatin treatment (2, 20 and 200 µM) led to a higher number of branching points in ECFCs after 6 h of incubation. A total of 2000 µM pravastatin, in contrast, reduced the number of branch points. (**C**) Pravastatin treatment (2, 20 and 200 µM) of ECFCs significantly increased tube length after 6 h of incubation, whereas 2000 µM pravastatin significantly impaired tube formation ability. Mevalonate (200 µM) did not affect tube formation ability in ECFCs but diminished pravastatin induced effects. In comparison to pravastatin treatment, only tube length was significantly less after pravastatin (2 µM, 20 µM and 200 µM) treatment in the presence of mevalonate. Mevalonate (200 µM) also attenuated the inhibitory effect on tube formation of high-dose pravastatin (2000 µM). Additional representative images of combined treatment can be found in Supplemental Figure S2. *n* = 12. Con, control; Prava, pravastatin; Mev, mevalonate. * *p* < 0.05, ** *p* < 0.01, *** *p* < 0.001 compared to control, ## *p* < 0.01 compared to 2 µM Prava, °°° *p* < 0.001 compared to 20 µM pravastatin, + *p* < 0.05 compared to 200 µM pravastatin, ^xx^ *p* < 0.01 compared to 2000 µM pravastatin; control group set as 1.

Reflecting their ability of de novo vessel formation in vivo, we performed an in vitro angiogenesis assay to determine pravastatin effects on ECFCs’ ability to form capillary-like structures in Matrigel. After treatment with low and intermediate doses of pravastatin, ECFCs showed significantly higher tube length than controls (2 µM: 1.21 ± 0.05, *p* = 0.009; 20 µM: 1.44 ± 0.08, *p* < 0.001; 200 µM: 1.37 ± 0.09, *p* < 0.001, *n* = 12) and higher numbers of branch points (2µM: 1.29 ± 0.09, *p* = 0.007; 20µM 1.75 ± 0.12; 200 µM: 1.40 ± 0.07, *p* < 0.001, *n* = 12), whereas a high dose of pravastatin led to a significant impairment of tube formation (relative tube length: 2000 µM: 0.47 ± 0.04; normalized number of branch points: 2000 µM: 0.44 ± 0.04, *n* = 12, *p* < 0.001), Figure 2A,B. Mevalonate had no significant impact on tube length (0.93 ± 0.09, *n* = 12, *p* = 0.42), but reduced or even reversed the positive pravastatin effect of lower and intermediate doses (2 µM: 0.97 ± 0.06, *p* = 0.30 compared to control and *p* = 0.007 compared to 2 µM pravastatin only; 20 µM: 0.97 ± 0.08, *p* > 0.99 compared to control and *p* < 0.001 compared to 20 µM pravastatin only; 200 µM: 1.12 ± 0.05, *p* = 0.02 compared to control and *p* = 0.02 compared to 200 µM pravastatin only, *n* = 12) and ameliorated the negative effect of the high pravastatin dose (2000 µM: 0.67 ± 0.04, *p* < 0.001 compared to control and *p* = 0.002 compared to 2000 µM pravastatin only, *n* = 12), Figure 2C, Supplemental Figure S2.

### 3.3. Pravastatin Has a Biphasic Impact on ECFC Proliferation 

**Figure 3:**
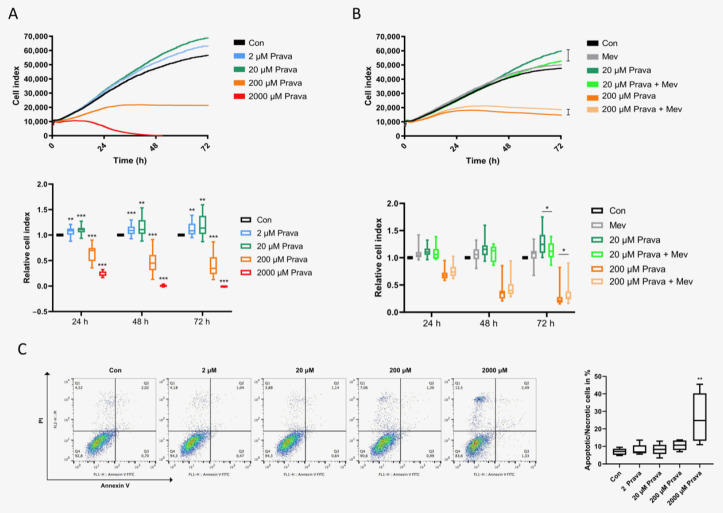
Pravastatin had a biphasic impact on ECFC proliferation. (**A**) Overlay of growth curves of ECFCs treated with pravastatin (2 µM, 20 µM, 200 µM or 2000 µM). ECFC proliferation was significantly increased after treatment with 2 µM or 20 µM pravastatin, but significantly decreased after treatment with 200 µM or 2000 µM pravastatin after 24 h, 48 h and 72 h. (**B**) Overlay of growth curves of ECFCs treated with 20 µM or 200 µM pravastatin in the presence or absence of 200 µM mevalonate. Mevalonate reduced the proliferative effect of 20 µM pravastatin and lessened the antiproliferative effect of 200 µM pravastatin significantly after 72 h. *n* = 15–20; control group set as 1. (**C**) High dose pravastatin led to apoptosis. Representative measurement of apoptosis and necrosis in ECFCs after 48 h treatment with pravastatin at 2 µM, 20 µM, 200 µM or 2000 µM. Viable cells are located in the lower left field (Annexin V neg./PI neg). Pravastatin (2000 µM) caused a significantly higher rate of non-viable cells. *n* = 5. Con, control; Prava, pravastatin; Mev, mevalonate. * *p* < 0.05, ** *p* < 0.01, *** *p* < 0.001.

Regarding one of their main characteristics, we addressed ECFCs’ proliferation in the presence or absence of different doses of pravastatin. The cell index was continuously monitored with the xCelligence technology. Treatment with 2 µM or 20 µM pravastatin significantly enhanced ECFCs’ proliferation (2 µM: 1.07 ± 0.02 after 24 h, *p* = 0.01, 1.10 ± 0.02 after 48 h *p* = 0.001, 1.12 ± 0.03 after 72 h, *p* = 0.001, *n* = 18; 20 µM: 1.10 ± 0.02 after 24h, *p* < 0.001, 1.15 ± 0.04 after 48 h, *p* = 0.002, 1.19 ± 0.05 after 72h, *p* = 0.001, *n* = 20), whereas treatment with 200 µM pravastatin attenuated cell growth (0.64 ± 0.04 after 24 h, 0.45 ± 0.05 after 48 h, 0.39 ± 0.05 after 72h, *p* < 0.001, *n* = 20) and led to a stable plateau after 24 h. The presence of 2000 µM pravastatin inhibited proliferation completely (0.23 ± 0.01 after 24 h, 0.01 ± 0.004 after 48 h, 0 ± 0.002 after 72 h, *p* < 0.001, *n* = 16), Figure 3A. The addition of mevalonate (200 µM) slightly lowered the increase in proliferation after treatment with 20 µM pravastatin only (1.13 ± 0.04 after 72 h, *n* = 15, *p* = 0.04 compared to 20 µM pravastatin: 1.28 ± 0.06) and moderately improved the impeded proliferation in the presence of 200 µM pravastatin (0.38 ± 0.05 after 72 h, *n* = 20, *p* = 0.03 compared to 200 µM pravastatin: 0.31 ± 0.05), Figure 3B. To determine whether apoptosis is involved in functional defects observed at higher pravastatin doses, we determined the expression level of the apoptosis marker annexin V using flow cytometry. After 48 h of incubation, there was no difference in apoptosis or necrosis in ECFCs treated with 2 µM (8.02% ± 1.43%, *n* = 5, *p* > 0.99), 20 µM (8.28% ± 1.52%, *n* = 5, *p* > 0.99) or 200 µM pravastatin (10.64% ± 1.26%, *n* = 5, *p* = 0.38) compared to the control (6.94% ± 0.83%). In accordance with the impaired proliferation at high pravastatin levels, we found a 3.8-fold increase in apoptosis while treated with 2000 µM pravastatin (26.35% ± 6.33%, *n* = 5, *p* = 0.007), Figure 3C. Mevalonate, addressed separately, did not induce any change in cell viability (0.97% ± 2.59%), presented as difference of means ± standard error of mean compared to the control (*p* = 0.72).

### 3.4. Pravastatin Enhances AKT and eNOS Phosphorylation and Increases HO-1 Expression

**Figure 4:**
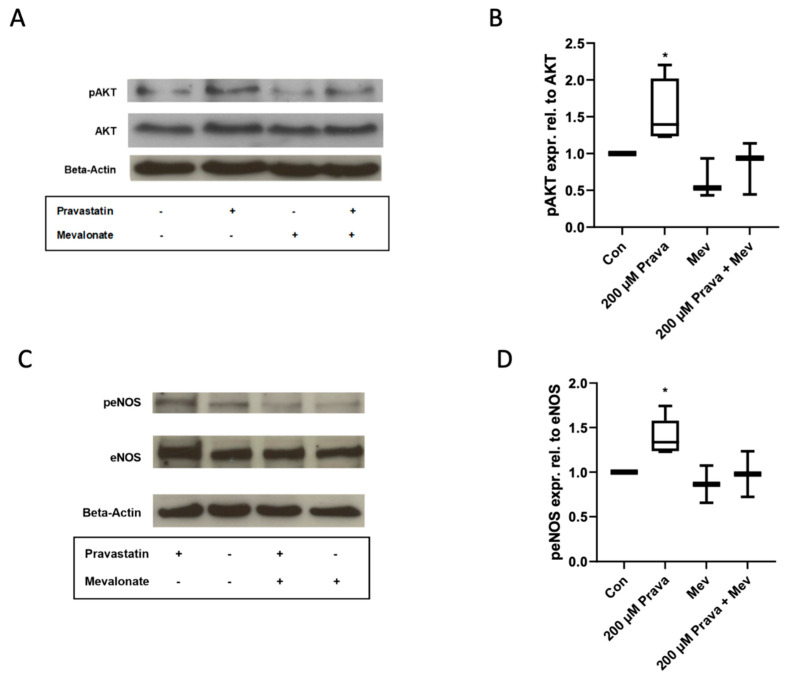
Pravastatin increased phosphorylation of protein kinase B (AKT) and endothelial nitric oxide synthase (eNOS). (**A**) Representative immunoblot of AKT phosphorylation after treatment with pravastatin (200 µM) in the presence or absence of mevalonate (200 µM). (**B**) Pravastatin significantly induced AKT phosphorylation, whereas mevalonate abrogates the pravastatin effect. (**C**) Representative immunoblot of eNOS phosphorylation after treatment with 200 µM pravastatin with or without the addition of mevalonate. (**D**) eNOS phosphorylation was significantly induced after incubation with pravastatin. Mevalonate reversed the pravastatin effect. The effect of pravastatin was investigated in 5 and of mevalonate at least in 2 independent experiments. Con, control; Prava, pravastatin; Mev, mevalonate. * *p* < 0.05; control group set as 1.

As the AKT-cascade is considered one of the most important signaling pathways in endothelial cells, we addressed possible pravastatin effects on AKT and its downstream target eNOS in ECFCs. Pravastatin (200 µM) induced AKT (1.57 ± 0.17, *n* = 6, *p* = 0.02, Figure 4A,B) and eNOS phosphorylation (1.39 ± 0.09, *n* = 5, *p* = 0.01, Figure 4C,D), while both effects were reduced by mevalonate (pAKT: 0.84 ± 0.21, *n* = 3, *p* = 0.52; peNOS: 0.98 ± 0.26, *n* = 2, *p* = 0.95). 

**Figure 5:**
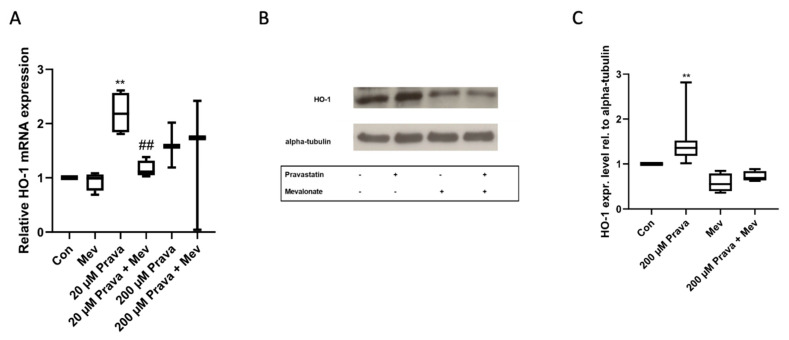
Pravastatin increased heme oxygenase-1 (HO-1) expression in real time PCR and immunoblot. (**A**) Pravastatin (20 µM) significantly induced HO-1 mRNA expression. The addition of mevalonate (200 µM) significantly reduced the pravastatin induced HO-1 mRNA expression. The increase in mRNA expression at intermediate pravastatin concentrations (200 µM) was not modulated by mevalonate at all. (**B**) Representative immunoblot of HO-1 protein expression after pravastatin (200 µM) treatment with or without additional mevalonate (200 µM). (**C**) Pravastatin significantly increased HO-1 protein expression in ECFCs, whereas mevalonate reversed the pravastatin induced effect. Real time PCR was performed in triplicates for at least 2 independent runs. Immunoblots were performed at least 4 times. Con, control; Prava, pravastatin; Mev, mevalonate. ** *p* < 0.01 compared to control, ## *p* < 0.01 compared to 20 µM pravastatin; control group set as 1.

We further tested whether pravastatin induces HO-1 expression level. There was an increase in HO-1 mRNA expression level in ECFCs after 24 h treatment with pravastatin (20 µM: 2.20 ± 0.19, *n* = 4, *p* = 0.009; 200 µM: 1.60 ± 0.24, *n* = 3, *p* = 0.13). The presence of mevalonate reduced the effect significantly for 20 µM pravastatin (1.16 ± 0.08, *n* = 4, *p* = 0.13 compared to control and *p* = 0.003 compared to 20 µM pravastatin only), but not consistently for 200 µM pravastatin (1.40 ± 0.71, *n* = 3, *p* = 0.68 compared to control and *p* = 0.81 compared to 200 µM pravastatin only), Figure 5A. 

In accordance with the increased HO-1 mRNA level observed in qRT-PCR after pravastatin treatment, there was also a higher HO-1 protein expression level (1.49 ± 0.20, *n* = 8, *p* = 0.008) in ECFCs after 24 h incubation with 200 µM pravastatin, shown by immunoblot analysis. The addition of mevalonate abrogated the pravastatin effect (0.72 ± 0.06, *n* = 4, *p* = 0.13), whereas mevalonate alone decreased the HO-1 expression level (0.58 ± 0.10, *n* = 4, *p* = 0.13), Figure 5B,C.

### 3.5. Pravastatin Affects mRNA and Protein Expression of Pro-Angiogenic and Anti-Angiogenic Factors 

**Figure 6:**
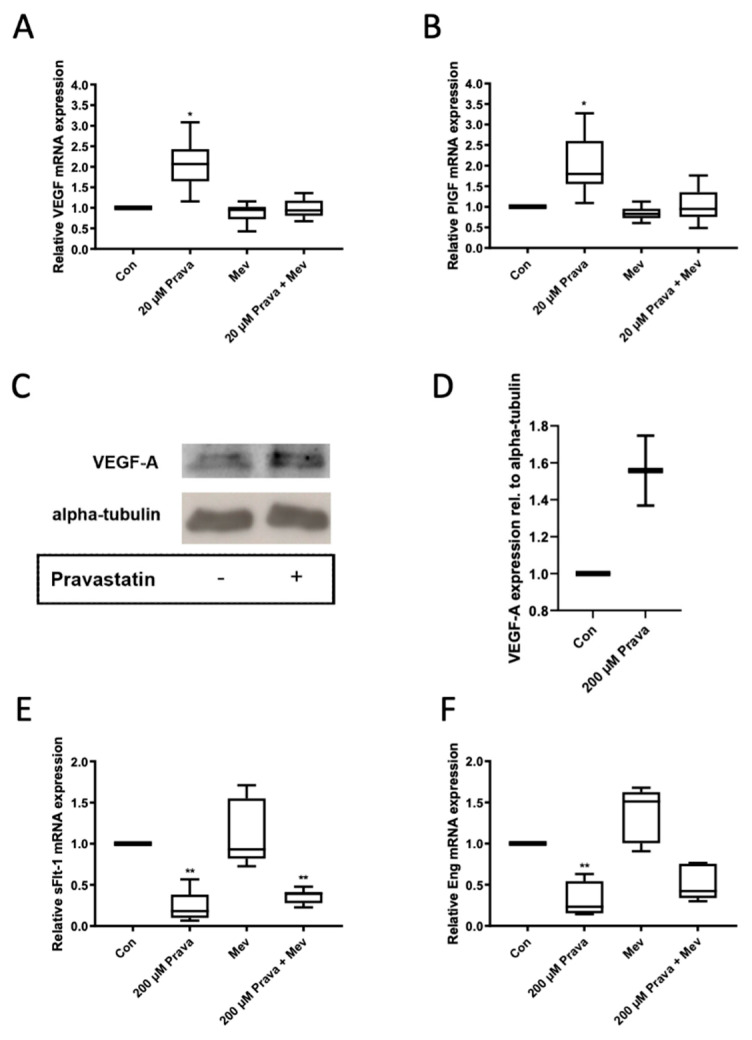
Pravastatin affected mRNA expression of pro-angiogenic and anti-angiogenic molecules. Pravastatin (20 µM) significantly induced mRNA expression of pro-angiogenic molecules vascular endothelial growth factor A (VEGF-A) (**A**) and placental growth factor (PlGF) (**B**), whereas mevalonate alone had no effect. In immunoblot (**C**), treatment with pravastatin (200 µM) increased VEGF-A protein expression (**D**). Co-treatment with mevalonate (200 µM) reduced pravastatin induced VEGF-A and PlGF mRNA expression. mRNA expression of the anti-angiogenic molecule soluble fms-like tyrosine kinase-1 (sFlt-1) (**E**) and the angiogenesis-related protein endoglin (Eng) (**F**) was significantly reduced by pravastatin (200 µM). Mevalonate (200 µM) did not significantly affect sFlt-1 mRNA expression but, in combination with pravastatin, reversed the pravastatin effect on sFlt-1 mRNA expression in ECFCs. Results are from at least 3 independent runs. All runs were performed in triplicates. Con, control; Prava, pravastatin; Mev, mevalonate. * *p* < 0.05, ** *p* < 0.01; control group set as 1.

To explore the mechanisms underlying the pro-angiogenic effects of pravastatin on ECFCs, we determined VEGF-A and PlGF mRNA levels after 24 h of treatment with pravastatin by using qRT-PCR and immunoblot. We also assessed mRNA expression levels of the anti-angiogenic factor sFlt-1 and the angiogenesis-related protein Eng. ECFCs that were treated with pravastatin (20 µM) showed a 2.03-fold higher expression level of VEGF-A (2.03 ± 0.27, *n* = 5, *p* = 0.02), Figure 6A, and a 2.1-fold higher expression level of PlGF (2.10 ± 0.34, *n* = 5, *p* = 0.03), Figure 6B, than the controls. The addition of mevalonate reversed the pravastatin effect (VEGF-A: 0.99 ± 0.10, *n* = 4, *p* = 0.89; PlGF: 1.02 ± 0.20, *n* = 4, *p* = 0.93). Regarding the protein expression, we observed a 1.56-fold higher VEGF-A expression after 24 h treatment with pravastatin (200 µM) (1.56 ± 0.19, *n* = 2, *p* = 0.21), Figure 6C,D. sFlt-1 and Eng mRNA expression levels were significantly reduced in the presence of 200 µM pravastatin (sFlt-1: 0.23 ± 0.09, *n* = 4, *p* = 0.003; Eng: 0.31 ± 0.11, *n* = 4, *p* = 0.008), Figure 6E,F. Mevalonate tended to lessen this effect (sFlt-1: 0.36 ± 0.05, *n* = 3, *p* = 0.007; Eng: 0.51 ± 0.13, *n* = 3, *p* = 0.06); however, sFlt-1 and Eng mRNA expression levels remained lower than the control.

## 4. Discussion

In the present study, we systematically explored pravastatin effects on human ECFC biology on functional and mechanistic levels. Our results reveal a significant promoting effect of low and moderate pravastatin concentrations on ECFCs’ functional capacities and further on AKT, eNOS and HO-1 signaling. However, pravastatin at high doses led to functional impairment and increased apoptosis and cell death. Specifically, we observed enhanced proliferative, migratory and angiogenic abilities of this EPC subtype and an augmented AKT and eNOS phosphorylation. Expression levels of HO-1 and the angiogenic molecules VEGF-A and PlGF were increased, whereas anti-angiogenic factor sFlt-1 and angiogenesis-related protein Eng expression levels were decreased. Pravastatin induced effects were diminished by adding the HMG-CoA reductase product mevalonate.

Endothelial progenitor cells have been addressed in several studies for more than 20 years [7]. Their numbers rise in response to tissue ischemia and they are able to attenuate endothelial dysfunction [54,55,56]. Endothelial colony-forming cells, also known as late-outgrowth EPC in culture, are a vasculogenic subgroup of EPCs and are considered the key group capable of vascular repair [23,57]. They are considered the most rational and promising cell source that is able to directly incorporate into or directly form regenerating vessels in areas of tissue regeneration [58]. Although there are excellent results in pre-clinical animal studies proving hope for patients with impaired vascular function, there are no reports of ECFCs application in human clinical trials yet [29]. As there is still not enough progress to integrate ECFCs in clinical practice, we investigated whether pravastatin may potentiate ECFCs’ numbers and functional capacities in vitro. Pravastatin, a HMG-CoA reductase inhibitor, is effective in the primary and secondary prevention of coronary heart disease [31,33]. It exerts extra-beneficial pleiotropic effects beyond the reduction in blood cholesterol levels, e.g., by the inhibition of inflammatory processes and oxidative stress in vessel walls [59].

We showed that pravastatin enhances ECFC migration towards a chemoattractive focus as well as in wound closure. This enhancement may also be beneficial regarding ECFCs’ homing to areas of endothelial damage in vivo. Statins’ favorable effects on cell migration have been shown in early-outgrowth EPCs, where they led to improved migration to VEGF producing foci [60]. In addition, we demonstrated that pravastatin improved ECFCs’ capacity of tube formation in vitro. In contrast to mature endothelial cells, ECFCs display the potential for revascularization after injection in vivo [25,61]. The ability of ECFCs to participate in neoangiogenesis opens up the possibility for the treatment of impaired wound healing in patients with diminished angiogenic capabilities [27]. However, high dose pravastatin treatment abolished ECFCs’ capacity of in vitro tube formation. These findings are consistent with previous reports of impaired angiogenesis in early outgrowth EPCs and mature endothelial cells at higher statin concentrations. Both studies attribute increased apoptosis as a reason for the observed alterations, which is in line with the higher proportion of apoptotic and necrotic cells we found in flow cytometry analysis [62,63]. 

Assmus et al. referred that the statin induced rise of EPC numbers after statin treatment in vitro might also improve their capacity in cell-based therapy [64,65]. In our study, we discovered a biphasic effect of pravastatin on ECFC proliferation. While lower and intermediate concentrations increased ECFC numbers, higher concentrations led to arrest and cell death in vitro. This biphasic effect has also been described by Nakao et al. in rat aortic endothelial cells and by Hu et al. in human cardiac microvascular endothelial cells [40,66].

There is a big bandwidth in literature of statin concentrations used in experimental studies [40,63,67]. The concentrations we used were based on previous publications of Brownfoot et al., who successfully investigated the effects of different statins on human umbilical vein endothelial cells and the placenta [67,68]. We started with a wide range for functional assays but then adjusted the range to the most effective ones according to self-performed pre-tests for the signaling analyses. As the concentrations we used are mostly higher than generally observed in blood plasma, our results mainly qualify for ex vivo cell therapy and might require adjustment in further in vivo studies.

Nearly all our observed effects were completely or partly reversed by the addition of mevalonate. Mevalonate is the product of the HMG-CoA reductase catalyzed reaction, which is inhibited by the application of statins. Cells require mevalonate, for example, for cholesterol-biosynthesis, isoprenylation and ubiquinone, which are involved in biomembrane composition, signal transduction and mitochondrial respiratory chain mediated energy production [69]. This may explain why high doses of pravastatin in our study led to a decrease in cell proliferation and higher rates of cell death and apoptosis. We also observed that the negative effects of high dose pravastatin became especially apparent in assays with a longer observation time, so there might also be a certain delay in functional and metabolic impairment due to a lack of ATP.

In endothelial cells, the majority of growth factor-induced reactions are considered to depend on AKT activation [70]. We therefore targeted AKT in our study and demonstrated a stimulating effect of pravastatin on AKT phosphorylation in ECFCs. AKT enhances cell migration to the growth factor producing focus [71], which is supported by our results obtained in the boyden chamber assay. Its activation is considered responsible for beneficial effects of statins, including postnatal neovascularization, more collaterals and higher capillary density in peripheral ischemia and an increase in EPC levels in mice [64,69,72,73].

Endothelial NOS is a downstream target in the AKT pathway. eNOS activity underlies complex regulatory processes and is determined, among other things, by different phosphorylation patterns [43]. We demonstrated a pravastatin induced increase in eNOS phosphorylation at Serine 1177 (Ser1177) in ECFCs, which was addressed because of its association with an activation of eNOS [74]. A diminished phosphorylation at Ser1177, however, has been observed in coronary heart disease, atherosclerosis and diabetes, and contributes to endothelial dysfunction [43]. The absence of eNOS is related to a severe reduction in the functional properties and viability of EPCs. Vice versa, eNOS activation by therapeutic intervention contributes to improved EPC induced neovascularization and viability after transfer into ischemic tissue [75,76,77,78]. An improvement in endothelial dysfunction in hypertensive rats was achieved by statin therapy through eNOS phosphorylation at Ser1177 [79].

As we have shown that the addition of mevalonate not only abrogates the functional effects of pravastatin on ECFCs, but also the pravastatin-induced phosphorylation of AKT and eNOS, it is likely that this pathway is mainly involved in pravastatin effect mediation. Inhibited statin-induced AKT-activation by the addition of mevalonate was also reported by Nakao et al., whereas Rossoni et al. described a mevalonate-inhibitable phosphorylation of eNOS in animal models [40,80].

Increased HO-1 gene and protein expression levels in ECFCs after pravastatin treatment have been detected in our study. HO-1 activation provides cell protection against oxidative stress and apoptosis and further maintains cell homeostasis in vitro and in vivo [41,81,82,83]. Transduction of late-outgrowth EPCs with AKT and HO-1 improved directional migration and neovascularization in mice after myocardial infarction [84]. Brownfoot et al. reported that pravastatin significantly induced HO-1 mRNA expression in human umbilical vein endothelial cells (HUVECs) [67]. HO-1 mRNA is stabilized via the AKT pathway [85] and HO-1 is considered necessary for the angiogenic function of EPCs. Its blockade is associated with reduced local expression of the pro-angiogenic growth factors VEGF and PlGF [86]. 

In our study, we found that pravastatin induced mRNA expression levels of VEGF-A and PlGF in ECFCs. This finding is consistent with a study in early-outgrowth EPCs, where pravastatin increased VEGF mRNA expression [45]. Further, we detected the tendency of an increase in VEGF-A protein expression. VEGF stimulates endothelial and progenitor cell migration and proliferation and mediates vascular growth and angiogenesis, whereas disruption of the VEGF/PlGF pathway leads to abnormal blood vessel development and further impedes ECFC-induced tubulogenesis [36,42,69,87,88]. Dubois et al. reported that ECFCs support cardiac neovascularization by releasing PlGF [89]. With regard to this paracrine effect of ECFCs, further research is required to analyze the pravastatin effect not only on intracellular transcription, but also on protein secretion.

Additionally, we demonstrated that pravastatin reduced the mRNA expression level of sFlt-1 and Eng, whose soluble form is considered to have anti-angiogenic properties. sFlt-1 blocks the binding of VEGF and PlGF to their receptors and has further been reported to inactivate eNOS [48]. An excess of sFlt-1 is associated with endothelial dysfunction in chronic kidney disease and it is suggested that increased sFlt-1 may predict cardiovascular risk [90]. Whereas the soluble form of Eng is associated with endothelial dysfunction and cardiovascular alterations, membrane bound Eng is related to vascular remodeling and angiogenesis [91]. Studies addressing statin effects on Eng expression have shown controversial results. In mice, on the one hand, endothelial expression of Eng is upregulated by hypercholesterolemia and decreased by statin treatment, suggesting an involvement of Eng in the process of atherogenesis [92]. On the other hand, an endoglin mediated beneficial effect on HUVECs via eNOS induction after statin treatment in vitro has been reported [93]. Changes of sEng levels might be related to membrane Eng expression; however, clear evidence of this correlation is still missing [94].

A disbalance of angiogenic and anti-angiogenic factors is also involved in the pathophysiology of preeclampsia with higher sFlt-1 as well as sEng concentrations in the circulation of women that develop the disease [49,95,96,97,98]. As our focus lies especially in gestational diseases, we decided to study the effect of pravastatin out of all statins because of its high hydrophilicity and, consequently, limited transplacental transfer [99]. Although statins are currently contraindicated in pregnancy [100], pravastatin is, in contrast to other statins, not considered teratogen and it has further been reported that pravastatin does not affect placental function [101,102,103]. Rodent studies have shown that pravastatin favorably influences angiogenic and anti-angiogenic factor expression in primary endothelial cells and the placenta and further ameliorates preeclampsia symptoms [44,47,67]; however, statin effects on sEng secretion were reported contradictory and require further investigation [104]. Pravastatin has recently been suggested as the statin of choice for reducing preeclampsia-associated endothelial dysfunction [105]. Further, HO-1 has been discussed as a target in the treatment of preeclampsia. Genetic studies established that HO-1 may prevent sFlt-1 and sEng overexpression of endothelium. A substance with the potential to activate the HO-1 system and to decrease sFlt-1 and sEng at the same time would possibly have favorable effects in preeclampsia [46,100]. In our study, we report both an increase in HO-1 and a reduction in sFlt-1 and Eng mRNA expression levels in ECFCs. This might be especially intriguing considering the fact that we have previously shown an impairment of ECFC biology in preeclampsia [17]. In randomized clinical trials, it is currently being tested whether pravastatin treatment can reduce the onset of preeclampsia in high-risk patients [106,107]. An increase in ECFCs’ number after eight weeks of pravastatin treatment has been reported in the peripheral blood of healthy postmenopausal women [108], but whether pravastatin treatment in pregnancy to reduce preeclampsia risk is associated with a rise in ECFC number and an improvement in ECFC function is not known and should be targeted in further studies as the meaningfulness of in vitro studies is important but limited, especially regarding possible effects of long-term exposition and concentration variances.

## 5. Conclusions

To our knowledge, this is the first study that systematically analyzed the effects of pravastatin on human ECFCs and elucidated involved signaling mechanisms. Our findings are of clinical relevance for various reasons. Firstly, our observations contribute to deepen the understanding of statins’ pleiotropic effects. As ECFCs have been shown to be impaired in several cardiovascular diseases, our findings might provide another approach to restore ECFCs’ function as a prognostic target in the future. Secondly, we further identified pravastatin treatment as a potential strategy to enhance ECFCs’ numbers and capacities prior to cell therapy and prepare the ground for further research regarding experimental animal models, e.g., of hindlimb ischemia or acute myocardial infarction, in order to ameliorate tissue damage via neovascularization and regeneration. Thirdly, these data add new supporting evidence to the current efforts in the development of preventative approaches with regards to preeclampsia and statin treatment.

## Figures and Tables

**Table 1:** Primer sequences for target genes.

Gene	Sense	Antisense
*VEGF-A*	TACCTCCACCATGCCAAGTG	GATGATTCTGCCCTCCTCCTT
*PlGF*	CCTACGTGGAGCTGACGTTCT	CCTTTCCGGCTTCATCTTCTC
*sFlt-1*	CTGTCTTCCAGAAAGTGCATTCA	TCACCACGTTGTTCTCAGATAAAAG
*Eng*	ACCTTTGGTGCCTTCCTCAT	CAATCCCTCAGAGGCTTCAC
*HO-1*	TTTCAGAAGGGCCAGGTGAC	GGAAGTAGACAGGGGCGAAG
*RNA18S1*	ACATCCAAGGAAGGCAGCAG	TTTTCGTCACTACCTCCCCG

## Data Availability

The data presented in this study are available on request from the corresponding author.

## Supplementary Materials

The following are available online at https://www.mdpi.com/2077-0383/10/2/183/s1, Supplemental Figure S1. Mevalonate decreases directional ECFC migration and reduces pravastatin (2000 µM) induced increase in directional migration. Representative images of DAPI-stained migrated ECFCs after treatment with 2000 µM pravastatin with or without 200 µM mevalonate. Con, control; Prava, pravastatin; Mev, mevalonate. Scale bar 150 µm. Supplemental Figure S2. Mevalonate (200 µM) modulates pravastatin’s effect on ECFCs’ tube formation ability. Representative images of ECFCs’ tube formation after 6 h in the presence of medium only (a), mevalonate (b), 20 µM pravastatin (c), 20 µM pravastatin + 200 µM mevalonate (d), 2000 µM pravastatin (e) or 2000 µM pravastatin + 200 µM mevalonate (f). Scale bar 500 µm.
