# Supplementary material for: Pravastatin Promotes Endothelial Colony-Forming Cell Function, Angiogenic Signaling and Protein Expression In Vitro
Source: J Clin Med. 2021 Jan 6;10(2):183. doi: 10.3390/jcm10020183 (PMC7825508; doi:10.3390/jcm10020183)
Supplement: Supplementary file 1 [file jcm-10-00183-s001.pdf]

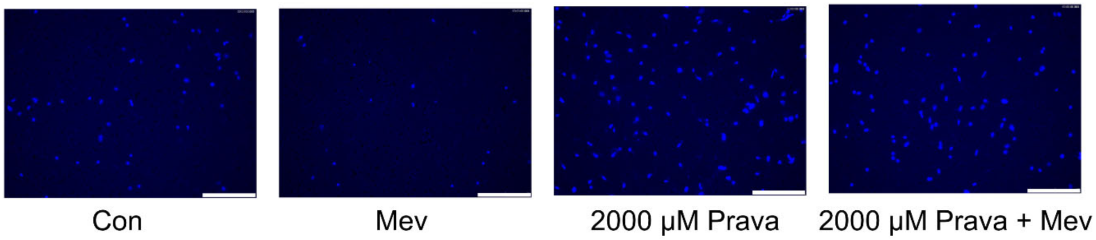

Supplemental Figure S1. Mevalonate decreases directional ECFC migration and reduces pravastatin (2000 μM) induced increase in directional migration. Representative images of DAPI-stained migrated ECFCs after treatment with 2000 μM pravastatin with or without 200 μM mevalonate. Con, control; Prava, pravastatin; Mev, mevalonate. Scale bar 150 μm.

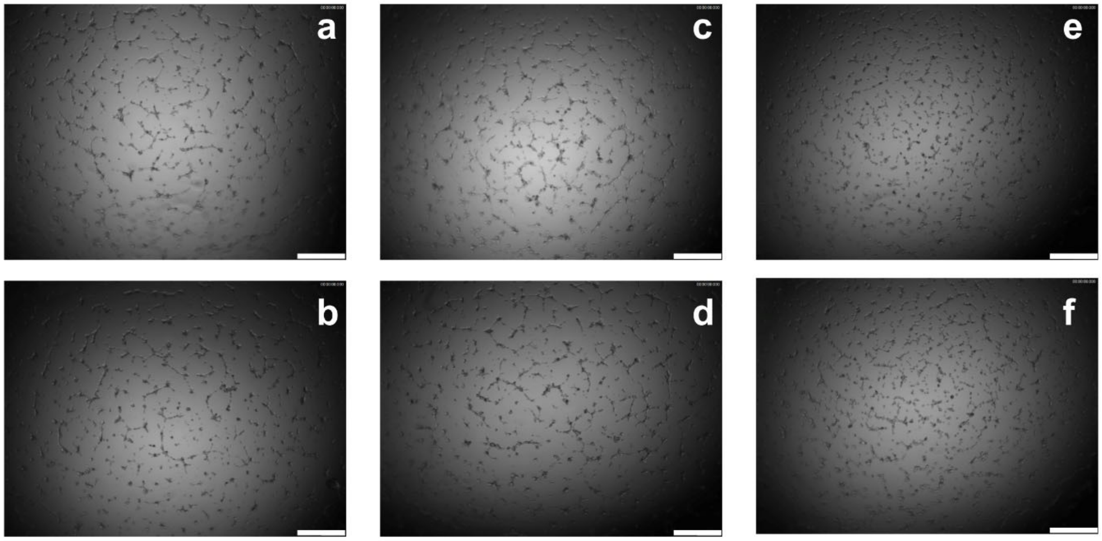

Supplemental Figure S2. Mevalonate (200 μM) modulates pravastatin's effect on ECFCs' tube formation ability. Representative images of ECFCs' tube formation after 6 h in the presence of medium only (a), mevalonate (b), 20 μM pravastatin (c), 20 μM pravastatin + 200 μM mevalonate (d), 2000 μM pravastatin (e) or 2000 μM pravastatin + 200 μM mevalonate (f). Scale bar 500 μm.
